# MiR-34a-Functionalized Hydroxyapatite by Lyophilization Promoted Bone Regeneration in Irradiated Bone Defects

**DOI:** 10.1155/2023/9946012

**Published:** 2023-09-11

**Authors:** Xi Wu, Xiaoke Feng, Gang Zhang, Huan Liu

**Affiliations:** ^1^Department of Stomatology, Xinqiao Hospital, Army Medical University, Chongqing 400037, China; ^2^Department of Prosthodontics, Tianjin Stomatological Hospital, School of Medicine, Nankai University, Tianjin 300041, China

## Abstract

The rehabilitation of bone defects after radiotherapy requires the development of osteoinductive bone substitutes. MicroRNA could be used as an osteogenic factor to fabricate functional materials for bone regeneration. In this study, we used miR-34a to enhance bone regeneration after irradiation. We lyophilized lipofectamine-agomiR-34a lipoplexes on hydroxyapatite (HA) to develop miR-34a-functionalized hydroxyapatite (HA-agomiR-34a). The morphology was observed by scanning electron microscope and atomic force microscope. Fluorescence microscopy confirmed the retention of agomiR-34a on the surface of HA. HA-agomiR-34a showed high transfection efficiency and good biocompatibility. HA-agomiR-34a enhanced the osteoblastic differentiation of radiation-impaired bone marrow stromal cells (BMSCs). Implantation of HA-agomiR-34a promoted bone regeneration in irradiated bone defects. HA-agomiR-34a may be a novel and safe bone substitute to promote the reconstruction of bone defects after radiotherapy.

## 1. Introduction

Patients with head and neck cancer often need combined treatment of surgery and radiotherapy [1]. Bone restoration is needed when resection results in extensive bone defects [2]. However, the restoration of large defects is often beyond the intrinsic regenerative potential and is impaired by radiotherapy [3]. Bone regeneration can be accomplished by using bone grafts, such as autografts, allografts, and alloplastic materials [4]. Hydroxyapatites (HA) have been successfully used for bone regeneration due to their excellent osteoconductive and osteointegrative properties. However, they do not possess osteoinductive properties [5, 6], which impedes their use in irradiated bone defects where the regenerative potential is impaired.

MicroRNA-based therapy is an advanced strategy in regenerative medicine. Synthetic microRNA (miRNA) mimics and inhibitors hold immense potential to regulate gene expression and reestablish tissue health [7]. MiR-34a is regarded as an efficient regulator of osteogenic differentiation and bone metabolism [8–10]. Our previous research found that miR-34a promoted the osteoblastic differentiation of bone marrow stromal cells (BMSCs) and enhanced bone healing after irradiation [11]. Besides, miR-34a-based therapy may prevent tumor recurrence [12]. Therefore, miR-34a may be used to enhance the osteoinductive properties of bone grafts to facilitate their use in the radiation-impaired area.

MicroRNA-based therapy requires simple and efficient delivery strategies [13]. Surface-mediated delivery refers to immobilizing nucleic acid-based therapeutics on a solid surface and delivering them to adjacent cells or the surrounding media [14, 15]. MiRNAs have been lyophilized on tissue culture plates [13] and titanium surfaces [16] to realize transfection and enhance the osteogenic differentiation of stem cells. Thus, lyophilization may be used to load miRNA on HA particles to enhance the osteoinductive property.

In this study, we lyophilized lipofectamine-miR-34a lipoplexes on HA particles to make miR-34a-functionalized HA, and we evaluated its transfection efficiency and biocompatibility. The osteoinductive property of HA-agomiR-34a under irradiated conditions was assessed in vitro and in vivo.

## 2. Materials and Methods

### 2.1. Study Design

MiR-34a agomiR (agomiR-34a), the negative control (agomiR-NC), and Cy3-labeled agomiR-34a (Cy3-agomiR-34a) were mixed with lipofectamine2000 (Invitrogen, USA) and lyophilized on hydroxyapatites (HA) to make HA-agomiR-34a, HA-agomiR-NC, and HA-Cy3-agomiR-34a. The morphology was observed by field-emission scanning electron microscope, atomic force microscope, and stereomicroscope. We used 2Gy X-ray-irradiated BMSCs for the in vitro study. Cells cultured with HA-Cy3-agomiR-34a were used for counting the transfection efficiency. Cells cultured with HA-agomiR-34a and HA-agomiR-NC were used for testing the expression of miR-34a. Cells cultured with HA-agomiR-34a, HA-agomiR-NC, and HA were used for the test of biocompatibility and osteoblastic differentiation. HA-Cy3-agomiR-34a was implanted in irradiated rat tibia bone defects to observe the in-situ delivery of miR-34a, and HA was used as a control. HA-agomiR-34a, HA-agomiR NC, and HA were implanted in irradiated rat tibia bone defects to study the function of HA-agomiR-34a on bone regeneration.

### 2.2. HA-agomiR-34a Preparation and Characterization

We used agomiR-34a, agomiR-NC, and Cy3-agomiR-34a (RiboBio, China) in this study. We dissolved 10 *μ*L lipofectamine2000 in 200 *μ*L of DEPC water and 500 pmol miRNA in 200 *μ*L of DEPC water and then mixed them together. The lipofectamine/miRNA complexes were mixed with 20 mg of HA (particles <200 nm, Sigma-Aldrich, USA) and frozen at −80°C for 2 hours, followed by 24 hours of lyophilization at −80°C to make HA-agomiR-34a, HA-agomiR-NC, and HA-Cy3-agomiR-34a. The morphology of HA-agomiR-34a was observed by a field-emission scanning electron microscope (SEM, Hitachi S-4800, Tokyo, Japan). Lipofectamine/agomiR-34a complexes (agomiR-34a/LP) lyophilized on the tissue culture plate surface and HA were used as a control. The morphology of HA-agomiR-34a was also observed by atomic force microscope (AFM, Agilent 5500, USA). AgomiR-34a/LP and HA dispersed in water were used as a control. HA-agomiR-34a was observed by fluorescence stereomicroscope (LEICA M205 FA, Wetzlar, Germany) for miRNA loading, and HA was used as a control.

### 2.3. Cell Culture, Cell Irradiation, and Osteoinduction

Two-week-old Sprague–Dawley rats were used for the isolation of BMSCs. The isolation procedure was described before [17]. Cells were cultured in *α*-minimum essential medium (*α*-MEM) supplemented with 10% fetal bovine serum (FBS; Sijiqing, Hangzhou, China) and 1% penicillin and streptomycin. The BMSCs were seeded in 12-well plates and irradiated with 2 Gy X-ray when reaching 95% confluency. Irradiation rate was set at 1.1 Gy/minute, kilovoltage at 160 kV, current at 25 mA, and source-surface distance at 50 cm (RS-2000 XE Biological Irradiator, Rad Source Technologies, GA, USA). The BMSCs were used 6 hours after irradiation.

To induce osteogenic differentiation, cells were cultured with osteogenic medium (10 mM *β*-glycerolphosphate, 50 *μ*g/ml Vc, and 10 nM dexamethasone, Sigma-Aldrich, USA). Intracellular ALP activity was tested by an ALP assay kit (Nanjing Jiancheng Bioengineering Institute, China) after 7 days of induction. Osteogenesis-related mRNA and protein expression were tested by quantitative real-time reverse transcription polymerase chain reaction (qRT-PCR) and Western blot after 14 days of induction. Three samples were included in each group at each time point (*n* = 3).

### 2.4. Transfection

We added 500 *μ*g HA-agomiR complexes to 12-well plates, and 1 × 10^5^ irradiated BMSCs were seeded into the wells. After 48 hours of transfection, cells were tested for miRNA uptake or used to induce osteogenic differentiation. Cy3-agomiR-34a was used to make HA-Cy3-agomiR-34a to observe the internalization of miRNAs by cells. 48 hours after transfection, cell nuclei were stained with Hoechst 33258 for 5 minutes. After washing three times with polybutylene succinate (PBS), the cells were observed using a confocal laser scanning microscope (Olympus FV1000, Tokyo, Japan), and cells cultured with HA were used as control. The transfection efficiency was detected by counting the percentage of Cy3-positive cells (*n* = 3). The expression of miR-34a in cells incubated with HA-agomiR-34a or HA-agomiR-NC was tested by qRT-PCR (*n* = 3). Cells incubated with HA-agomiR-34a, HA-agomiR-NC, and HA were used to induce osteogenic differentiation.

### 2.5. Cell Viability

Cells cultured with HA-agomiR-34a, HA-agomiR-NC, and HA were used to test the cell viability that was evaluated by cell counting kit-8 (CCK-8, Beyotime, China) after 48 hours of transfection and 7 days of osteogenic induction (*n* = 3). We replaced the culture medium with 500 *μ*L of fresh culture medium and 50 *μ*L of CCK-8 solution at the time point. After incubation at 37°C for 2 hours, 200 *μ*L of supernatant was transferred to a 96-well plate, and the absorbance was measured at 450 nm (Thermo LabSystems Beverly, USA).

### 2.6. Cell Morphology

Cells cultured with HA-agomiR-34a, HA-agomiR-NC, and HA were used for the observation of cell morphology. After 48 hours of transfection and 7 days of osteogenic induction, cells were washed with PBS, fixed in 2.5% glutaraldehyde, dehydrated in a graded ethanol series and freeze-dried, covered with gold, and observed with the SEM (Hitachi S-4800, Tokyo, Japan).

### 2.7. RNA Extraction and Quantitative Real-Time PCR (qRT-PCR)

Total RNA was extracted by TriZol (Invitrogen, USA). 500 ng RNA was used for reverse transcription with the PrimeScript RT reagent kit (TaKaRa, Japan). Quantitative real-time PCR was performed with SYBR PremixExTaqTMII (TaKaRa, Japan) on the CFX96 Real-Time RT-PCR System (Biorad, USA). Relative expression was calculated by the ΔΔCt method, and *gapdh* was used for normalization. The primers were synthesized as shown in Table 1. For miR-34a quantification, U6 was used for normalization. Bulge-loopTM qRT-PCR primer sets including reverse transcription primer and qPCR primers were designed by Ruibo.

### 2.8. Western Blot Analysis

Cells were lysed in RIPA buffer supplemented with a protease inhibitor cocktail (Sigma, MO, USA). Protein concentrations were quantified by the BCA protein assay (Beyotime, China), and 40 *μ*g protein of each sample was loaded on 10% SDS-PAGE gels and transferred to the PVDF membranes. The membranes were blocked with 5% BSA and incubated with primary antibodies RUNX2 (Santa Cruz Biotechnology, sc-10758), ALP (Protein tech, 11187-1-AP), osteocalcin (OCN; Santa Cruz Biotechnology, sc-390877), BMP2 (ABclonal, A0231), and GAPDH (Abcam, ab8245) at 4°C overnight. After a 2-hour incubation with secondary antibodies (Cowin Biotech, China), the bands were incubated with a chemiluminescence kit (Amersham Biosciences, USA) and visualized using the imaging system (Tanon 5500, China). The grey value of the protein bands was quantified using Image-Pro Plus 6.0 software and normalized to that of glyceraldehyde-3-phosphate dehydrogenase (GAPDH) before comparison.

### 2.9. Rat Tibial Defect Model

Rats were fixed in a perspex jig with their tibias extended laterally for irradiation, and other parts of the body were protected with lead shielding. The tibias of rats were subjected to a single dose of 15 Gy X-ray irradiation using an RS-2000 XE Biological Irradiator (Rad Source Technologies, GA, USA). The irradiation rate was set at 1.1 Gy/minute, kilovoltage at 160 kV, current at 25 mA, and source-surface distance at 50 cm. Bone defect surgeries were conducted 3 weeks after irradiation. A 3-mm defect was generated in both tibias. 20 mg HA-agomiR-34a, HA-agomiR NC, HA-Cy3-agomiR-34a, or HA were implanted within the defects.

To study the in-situ delivery of miR-34a, rats were sacrificed at 2, 4, and 8 weeks after surgery. For histological observation, HA-Cy3-agomiR-34a and HA were implanted. Tibias were fixed, decalcified, and cut into 10 *μ*m thick frozen sections. Sections were stained with Hoechst 33342 (Sigma-Aldrich) for 3 min and observed under a confocal microscope (Olympus FV1000, Tokyo, Japan). For the test of the miR-34a level in bone defects, HA-agomiR-34a and HA-agomiR-NC were implanted. The newly formed bone in the defect area was harvested and ground in liquid nitrogen (*n* = 3). The total RNA was extracted with Trizol reagent (Invitrogen, USA), and miRNA expression was evaluated by qRT-PCR.

To study the function of miR-34a-functionalized HA on bone repair, HA-agomiR-34a, HA-agomiR-NC, and HA were implanted. Bone regeneration in the defects was evaluated 8 weeks after implantation employing micro-CT, hematoxylin and eosin (H&E) staining, and sequential fluorescent labelling assay.

### 2.10. Test of Micro-CT

Tibias were fixed in 4% paraformaldehyde and scanned by micro-CT (Y.Cheetah, Y.XLON, Germany) at a resolution of 18 *µ*m. Data analysis was performed using VG StudioMAX (Volume Graphics, Germany). The region of interest (ROI) was the original bone defect area (*L*, 2 mm; *φ*, 3 mm). We calculated volume/total volume (BV/TV) to compare the bone regeneration (*n* = 6).

### 2.11. H&E Staining

Tibias were decalcified for four weeks in 18% EDTA (pH 7.0) and embedded in paraffin, and 5-*μ*m-thick sections were stained with hematoxylin and eosin (H&E).

### 2.12. Sequential Fluorescent Labelling Assay

Rats were injected with alizarin red S (30 mg/kg, Sigma), calcein (20 mg/kg, Sigma), and tetracycline hydrochloride (20 mg/kg, Sigma) at 3, 5, and 7 weeks after the surgery. Tibias were harvested eight weeks after surgery and embedded in polymethylmethacrylate (PMMA). Samples were cut into 50-*μ*m sections (LEICA SP1600, Wetzlar, Germany) and observed with a confocal laser scanning microscope (Olympus FV1000, Tokyo, Japan). Excitation/emission wavelengths of chelating fluorochromes were used 561/617 nm, 488/517 nm, and 405/580 nm for alizarin red S (red), calcein (green), and tetracyclin hydrochloride (yellow), respectively. The area of fluorescent labelling was quantified by Image-Pro Plus 6.0 software (*n* = 3).

### 2.13. Statistical Analysis

All experiments were repeated at least three times, and data were presented as mean ± SD. Differences between the two groups were analyzed by Student's *t*-test. Differences among groups were analyzed by one-way ANOVA followed by Tukey's posttest. GraphPad Prism 8 software was used, and *P* < 0.05 was considered significantly different.

## 3. Results

### 3.1. Characterization of miRNA Functionalized HA

**Figure 1:**
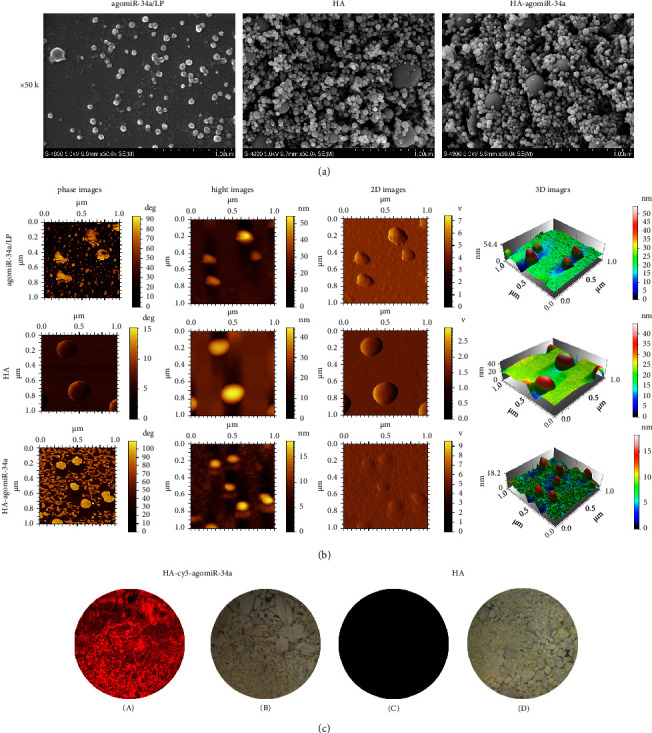
Characterization of miRNA functionalized HA. (a) Representative SEM images of agomiR-34a/LP, HA, and HA-agomiR-34a. (b) Representative AFM images of agomiR-34a/LP, HA, and HA-agomiR-34a. (c) HA-Cy3-agomiR-34a under fluorescence condition (A) and bright-field condition (B), HA under fluorescence condition (C), and bright-field condition (D).

The SEM images of agomiR-34a/LP, HA, or HA-agomiR-34a are shown in Figure 1(a). AgomiR-34a/LP lyophilized on the tissue culture plate surface was composed of pseudospherical particles. The diameters ranged from 40 nm to 200 nm. HA was composed of nanoparticles with sizes from 50 to 250 nm. The morphology of HA-agomiR-34a was similar to that of HA.

The AFM images of agomiR-34a/LP, HA, and HA-agomiR-34a dispersed in water showed that their morphology and sizes were consistent with those observed by SEM (Figure 1(b)).

The fluorescence images of HA-Cy3-agomiR-34a confirmed the retention of Cy3-agomiR-34a on the surface of HA (Figure 1(c)).

### 3.2. HA-agomiR-34a Upregulated the Expression of miR-34a in BMSCs

**Figure 2:**
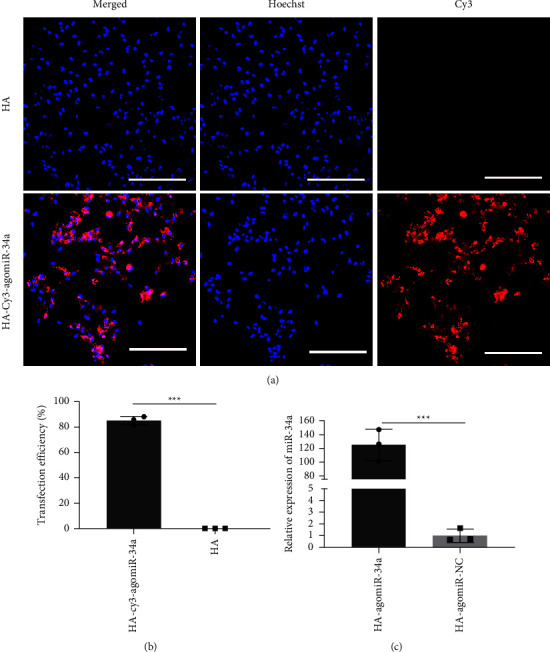
Transfection efficiency of HA-agomiR-34a. (a) Images of Cy3-positive 2 Gy irradiated BMSCs; scale bar = 200 *μ*m. (b) The histogram of Cy3-positive cell percentage. (c) MiR-34a expression determined by qRT-PCR in BMSCs 48 hours after transfection. Data are shown as mean ± SD, *n* = 3; ^*∗∗∗*^*p* < 0.001.

Fluorescence images showed that Cy3-agomiR-34a was located around the cell nuclei (Figure 2(a)). The transfection efficiency was about 80% (Figure 2(b)). The expression level of miR-34a of BMSCs cultured with HA-agomiR-34a was significantly higher compared with the HA-agomiR-NC group (Figure 2(c)). After being stored at 4°C for 90 days, HA-agomiR-34a could still efficiently enhance the expression level of miR-34a in BMSCs (Figure S1).

### 3.3. Biocompatibility of HA-agomiR-34a

**Figure 3:**
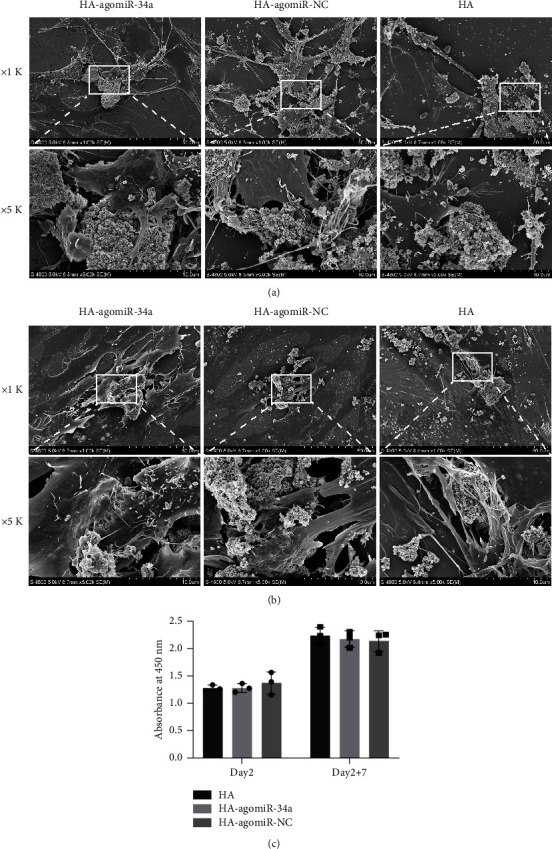
Biocompatibility of HA-agomiR-34a. (a) Representative SEM images of cells cultured with HA-agomiR-34a, HA-agomiR-NC, and HA 2 days after culture. (b) SEM images 7 days after osteogenic incubation. (c) Cell viability measured by CCK-8 2 days after culture (day 2) and 7 days after osteogenic incubation (day 2 + 7) (*n* = 3).

Cell morphology was observed by SEM. The morphological features of BMSCs were similar among different groups. 2 days after culture, BMSCs could attach to HA particles of each group and form abundant filopodia (Figure 3(a)). 7 days after osteogenic incubation, BMSCs were widely spread over the material with irregularly branched cytoplasm (Figure 3(b)).

The CCK-8 assay was used to measure cell viability; compared with HA, functionalization with HA-agomiR-34a or HA-agomiR-NC did not influence cell viability (Figure 3(c)).

### 3.4. HA-agomiR-34a Enhanced the Osteoblastic Differentiation of Irradiated BMSCs In Vitro

**Figure 4:**
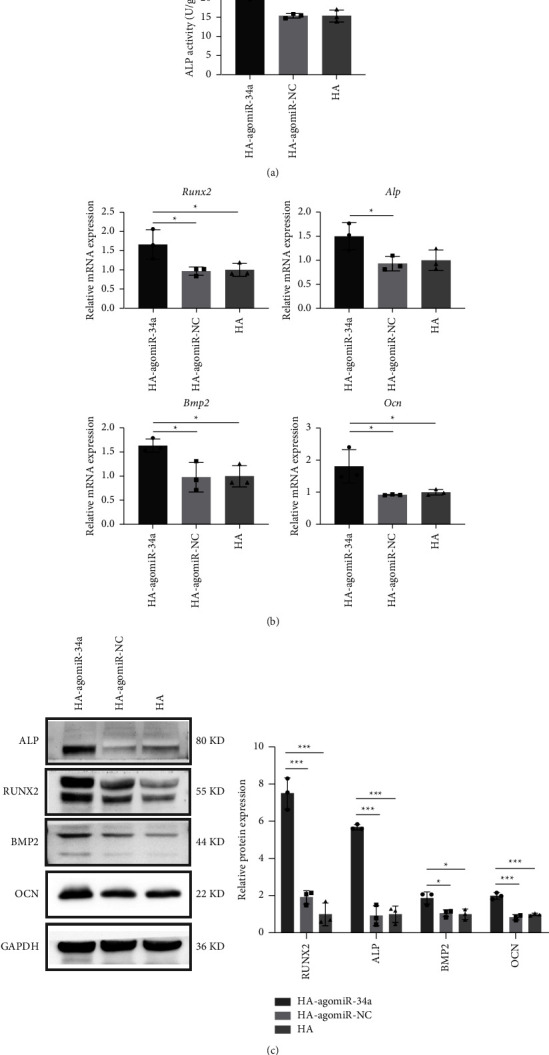
Osteogenic differentiation of 2 Gy-irradiated BMSCs in vitro. (a) Intracellular ALP activity after 7 days of osteogenic induction. (b) Gene expression of *Runx2*, *Alp*, *Bmp2*, and *Ocn* after 14 days of osteogenic induction. (c) Western blot of ALP, RUNX2, BMP2, OCN, and GAPDH after 14 days of osteogenic induction. Quantitative analysis of the Western blot results relative to GAPDH. Data are shown as mean ± SD, *n* = 3; ^*∗*^*p* < 0.05, ^*∗∗*^*p* < 0.01, ^*∗∗∗*^*p* < 0.001.

Irradiation with 2 Gy X-rays impairs the osteoblastic differentiation of BMSCs (Figure S2). To verify whether HA-agomiR-34a could enhance the osteoblastic differentiation of irradiated BMSCs in vitro, we cultured 2 Gy irradiated BMSCs with HA-agomiR-34a, HA-agomiR-NC, and HA. The Alp activity in the HA-agomiR-34a group was the highest among the three groups (Figure 4(a)). The expression of osteogenesis-related genes was examined by qRT-PCR (Figure 4(b)). The expression of *Runx2*, *Bmp2*, and *Ocn* was higher in the HA-agomiR-34a group than in the other two groups. The expression of *Alp* was higher in the HA-agomiR-34a group than in the HA-agomiR-NC group. The protein expression of osteogenic markers was assessed by Western blot (Figure 4(c)). The expression of RUNX2, ALP, BMP2, and OCN at the protein level was higher in the HA-agomiR-34a group than in the other two groups.

### 3.5. HA-agomiR-34a Downregulated the Expression of NOTCH1

**Figure 5:**
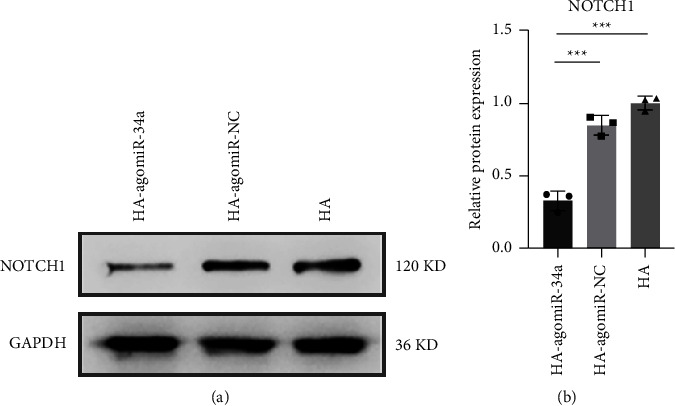
HA-agomiR-34a downregulated the expression of NOTCH1. (a) Western blot analysis of NOTCH1. (b) The quantitative analysis of the Western blot results relative to GAPDH. Data are shown as mean ± SD, *n* = 3; ^*∗∗∗*^*p* < 0.001.

NOTCH1 was previously identified as a target of miR-34a [8]. Our previous study confirmed that miR-34a promotes osteoblastic differentiation of irradiated BMSCs by regulating NOTCH1 [11]. In this study, we tested the expression of NOTCH1 in BMSCs after osteogenic induction. The protein expression of NOTCH1 was lower in the HA-agomiR-34a group than in the other two groups (Figure 5).

### 3.6. HA-agomiR-34a Upregulated the In Situ Expression Level of miR-34a

**Figure 6:**
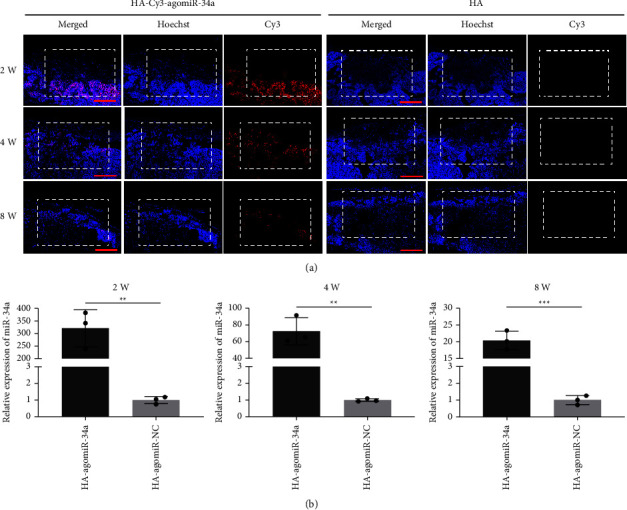
In-situ delivery of agomiR-34a. (a) Representative images of Cy3-labelled agomiR-34a in the bone defect area 2, 4, and 8 weeks after implantation; scale bar = 1 mm. The dotted boxes indicate the bone defects. (b) MiR-34a expression determined by qRT-PCR in the newly formed bone 2, 4, and 8 weeks after implantation. Data are shown as mean ± SD, *n* = 3; ^*∗∗*^*p* < 0.01, ^*∗∗∗*^*p* < 0.001.

After implantation of HA-Cy3-agomiR-34a in bone defects, Cy3 fluorescence was obvious at 2 weeks and decreased at 4 and 8 weeks (Figure 6(a)). The expression of miR-34a was about 300-fold higher in the HA-agomiR-34a group than in the control group 2 weeks after surgery. At 4 weeks or 8 weeks, the miR-34a level in the HA-agomiR-34a group showed a 60-fold or 20-fold increase over the control group (Figure 6(b)).

### 3.7. HA-agomiR-34a Enhanced Bone Formation in Irradiated Bone Defects

**Figure 7:**
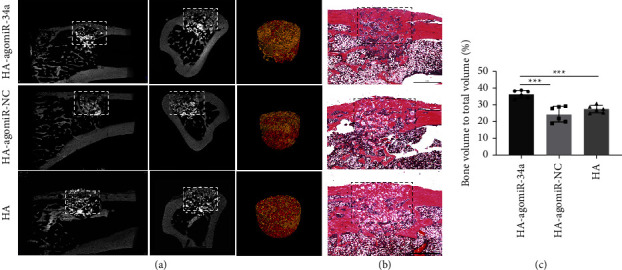
HA-agomiR-34a enhanced bone formation in irradiated bone defects. (a) 2D and 3D micro-CT images of bone formation in the defect area of irradiated rats at 8 weeks. The dotted boxes indicate the bone defects. The yellow parts represent the newly formed bone. The red parts represent the HA particles. (b) Representative H&E staining of new bone formation; scale bar = 1 mm. The dotted boxes indicate the bone defects. (c) The morphometric analysis of BV/TV for micro-CT images. Data are shown as mean ± SD, *n* = 6; ^*∗∗∗*^*p* < 0.001.

New bone formation in the defect was assessed by micro-CT 8 weeks after surgery. Bone healing could be observed in all three groups while HA particles remained. The volume of newly formed bone in the HA-agomiR-34a group was higher than in the other two groups (*p* < 0.001), and the continuity of cortical bone was restored. The volume of newly formed bone in the HA-agomiR-NC and HA groups was not significantly different. H&E staining confirmed the results of the micro-CT scanning (Figure 7).

**Figure 8:**
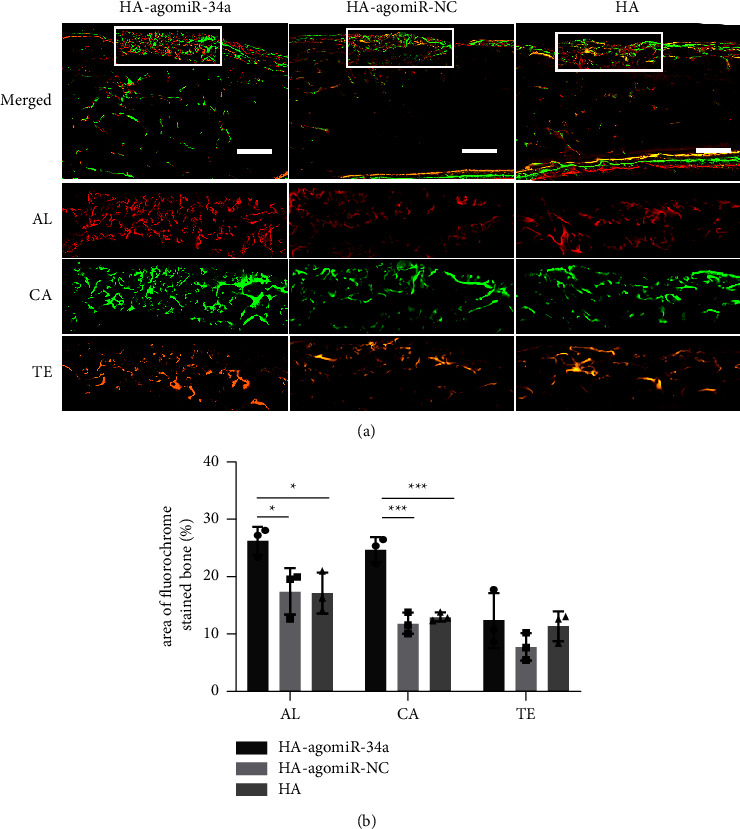
Sequential fluorescent labelling of bone formation and mineralization. (a) The upper panels show the overall image of each group. The lower panels show the area within the white boxes indicating new bone formed in the defect area. Alizarin red S (AL), calcein (CA), and tetracycline hydrochloride (TE); scale bar = 1 mm. (b) The quantitative results of sequential fluorescent labelling. Data are shown as mean ± SD, *n* = 3; ^*∗*^*p* < 0.05, ^*∗∗∗*^*p* < 0.001.

The area of fluorescent labelling was used to quantify the bone formation and mineralization (Figure 8). The HA-agomiR-34a group showed the highest percentage of fluorescent labelling at 3 and 5 weeks. At 7 weeks, there was no difference among the three groups.

## 4. Discussion

Developing osteoinductive bone substitutes that are effective under irradiated conditions is essential for the maxillofacial and oral rehabilitation of cancer patients [3, 18]. In this study, we lyophilized lipofectamine-miR-34a lipoplexes on HA particles to make miR-34a-functionalized HA. HA-agomiR-34a showed high transfection efficiency and adequate biocompatibility. Based on in vitro and in vivo analyses, we found that miR-34a-functionalized HA could improve bone regeneration after irradiation.

Radiotherapy has inhibitory effects on bone formation. BMSC is known to be the precursor of bone cells, and irradiation could impair the osteoblastic differentiation of BMSCs [19, 20]. Impaired bone healing, as manifested by woven bone, immature bone marrow, and decreased bone mineral density, has been reported in irradiated defects in rats [21]. In our previous research, we confirmed that 2 Gy X-ray irradiation impairs the osteoblastic differentiation of BMSCs, and 15 Gy X-ray irradiation caused a delay in the osseous closure [11]. Here, we used the same irradiation method to assess the effect of miR-34a-functionalized HA on bone defect healing and found that HA-agomiR-34a could promote osteogenesis after irradiation. However, besides the delay in bone defect healing, osteoradionecrosis is a common complication of cancer radiotherapy in the head and neck [22]. Further research involving irradiated orofacial bones is necessary before considering the use of this new material after head and neck cancer treatment.

MiRNA-loaded biomaterials exhibit excellent biosafety and could effectively promote bone regeneration [23, 24]. Inorganic biomaterials such as metal materials [16, 25] and calcium orthophosphate [26, 27] could load miRNA by surface coating and provide mechanical support in bone defects. We used HA as the miR-34a loading material because it has a similar structure to bone and has been used for bone regeneration and drug delivery [28]. We prepared lipofectamine-miR-34a lipoplexes and mixed them with HA. The miR-34a lipoplexes could be loaded on HA by lyophilization, as shown by fluorescence images. Nucleic acid complexes need to remain small to allow cellular internalization from the material surface or the surrounding media [29]. SEM showed that, after lyophilization, agomiR-34a/LP was pseudospherical particles after lyophilization ranging from 40 nm to 200 nm. This result is consistent with Wu's report [13], meaning that the morphology of miR-34a lipoplexes was not destroyed by lyophilization. HA and HA-agomiR-34a were composed of nanosized particles ranging from 50 to 250 nm. When dispersed in water and observed by AFM, agomiR-34a/LP, HA, and HA-agomiR-34a displayed pseudospherical morphology with a diameter of 50 to 250 nm. These results confirmed that miR-34a lipoplexes could be lyophilized on HA and used for local delivery of miR-34a.

We found that HA-agomiR-34a could deliver miR-34a to cells and upregulate the expression of miR-34a within bone defects. In vitro, we observed that Cy3-labelled agomiR-34a is located around cell nuclei, and the expression level of miR-34a of BMSCs cultured with HA-agomiR-34a was significantly enhanced. In vivo, we found that the miR-34a level within the original bone defects was much higher in the HA-agomiR-34a group after implantation. We also found that BMSCs transfected by HA-agomiR-34a expressed higher levels of miR-34a than when transfected by the conventional way (Figure S3). HA-agomiR-34a has good transfection ability probably because of the high efficiency of reverse transfection. HA could deliver surface-bounded lipofectamine/agomiR-34a complexes directly to cells or to the surrounding media. This reverse transfection method combines the advantages of the lipoplexes carrier system and the surface-mediated delivery [30]. Other research has also shown that miRNA-functionalized material through lyophilization has high transfection activity [15]. Besides, as HA nanoparticles could be uptaken by cells [26, 31], HA-agomiR-34a may also deliver agomiR-34a intracellularly through uptake; this internalization of HA nanoparticles was observed under TEM in our study (Figure S4). Furthermore, HA-agomiR-34a maintained high transfection efficiency after being stored at 4°C for 90 days (Figure S1). These results confirmed that HA-agomiR-34a has good transfection ability and storability.

MiR-34a is an efficient regulator of bone metabolism [32], partly through modulating NOTCH1 [8]; it has been used to develop miRNA-loaded biomaterials. Guo loaded miR-34a in a hydroxyapatite/mesoporous organosilica nanoparticles composite-coated implant wire to accelerate bone fracture healing [33]. Shen combined N-Ac-L-Leu-PEI/miR-34a nanocomplexes with the gelatin sponge to promote new bone formation in rat cranial bone defects [9]. We used hydroxyapatite (HA) as the loading material. HA-agomiR-34a enhanced bone regeneration in irradiated bone defects possibly because miR-34a could promote the osteoblastic differentiation of irradiated BMSCs by downregulation of NOTCH1 (Figure 5), and HA could provide a favorable environment for cell attachment [34]. HA-agomiR-34a may be used as an independent bone-filling material or as a modifier for synthetic materials. Moreover, this miR-34a-functionalized HA may potentially be used in bone defects after cancer treatment because miR-34a is regarded as a promising therapeutic agent against cancer [35].

One concern in this study is the use of lipofectamine, because cationic liposomes, including lipofectamine 2000, exhibit toxicity [36]. However, controlling the amount of lipofectamine in the transfection formulation could achieve good cytocompatibility [13]. HA-agomiR-34a showed low cytotoxicity (Figure 3(c)), and BMSC adhered to HA-agomiR-34a particles via lamellipodia and filopodia (Figures 3(a) and 3(b)), indicating that HA-agomiR-34a possessed good biocompatibility and could provide a favorable environment for cell attachment. Moreover, lipofectamine may cause rapid clearance and high uptake by the liver and spleen [37] when delivered systemically. However, when delivered locally, as in this study, we found an elevated level of miR-34a in the regenerated bone 8 weeks after implantation. Another concern is the precision application [37]. We reported that miR-34a could promote the osteoblastic differentiation of irradiated BMSCs, but miR-34a also regulates osteoclasts [33, 38] and endothelial cells [39]. A BMSC-targeted design is needed to modify HA-agomiR-34a to achieve precision medicine.

## 5. Conclusions

In the present study, we demonstrated that lipofectamine-miR-34a lipoplexes could be lyophilized onto HA particles to make miR-34a-functionalized bone substitutes. HA-agomiR-34a showed high transfection efficiency, good long-term storability, and biosafety. HA-agomiR-34a promoted the osteoblastic differentiation of irradiated BMSCs and improved bone regeneration in bone defects of irradiated rat tibias. These findings indicated that HA-agomiR-34a might provide a potentially safe strategy to promote the reconstruction of the bone after radiotherapy.

## Figures and Tables

**Table 1:** Primers used for qRT-PCR.

Gene	Forward primer sequence (5′-3′)	Reverse primer sequence (5′-3′)
*Runx2*	5′ AGA CCA GCA GCA CTC CAT AT 3′	5′ CTC ATC CAT TCT GCC GCT AGA 3′
*Alp*	5′ ATG GCT CAC CTG CTT CAC G 3′	5′ TCA GAA CAG GGT GCG TAG G 3′
*Ocn*	5′ AGG GCA GTA AGG TGG TGA AT 3′	5′ GCA TTA ACC AAC ACG GGG TA 3′
*Bmp2*	5′ GAAGCCAGGTGTCTCCAAGA 3′	5′ GGATGTCCTTTACCGTCGT 3′
*Gapdh*	5′ GGCACAGTCAAGGCTGAGAATG 3′	5′ ATGGTGGTGAAGACGCCAGTA 3′

## Data Availability

All data that support the findings of this study are available from the corresponding author upon reasonable request.

## Supplementary Materials

Supplementary Materials Figure S1: Storable stability of miR-34a functionalized HA. Figure S2: The osteoblastic differentiation and proliferation of nonirradiated and 2 Gy-irradiated BMSCs. Figure S3: Comparison of miR-34a expression between HA-agomiR-34A-mediated transfection and conventional transfection. Figure S4: The internalization of nanoparticles.
