# Supplementary material for: MiR-34a-Functionalized Hydroxyapatite by Lyophilization Promoted Bone Regeneration in Irradiated Bone Defects
Source: J Tissue Eng Regen Med. 2023 Sep 11;2023:9946012. doi: 10.1155/2023/9946012 (PMC11918688; doi:10.1155/2023/9946012)
Supplement: Supplementary Materials — Figure S1: Storable stability of miR-34a functionalized HA. Figure S2: The osteoblastic differentiation and proliferation of nonirradiated and 2 Gy-irradiated BMSCs. Figure S3: Comparison of miR-34a expression between HA-agomiR-34A-mediated transfection and conventional transfection. Figure S4: The internalization of nanoparticles. [file 9946012.f1.docx]

**Additional file 1**

**Figure S1**


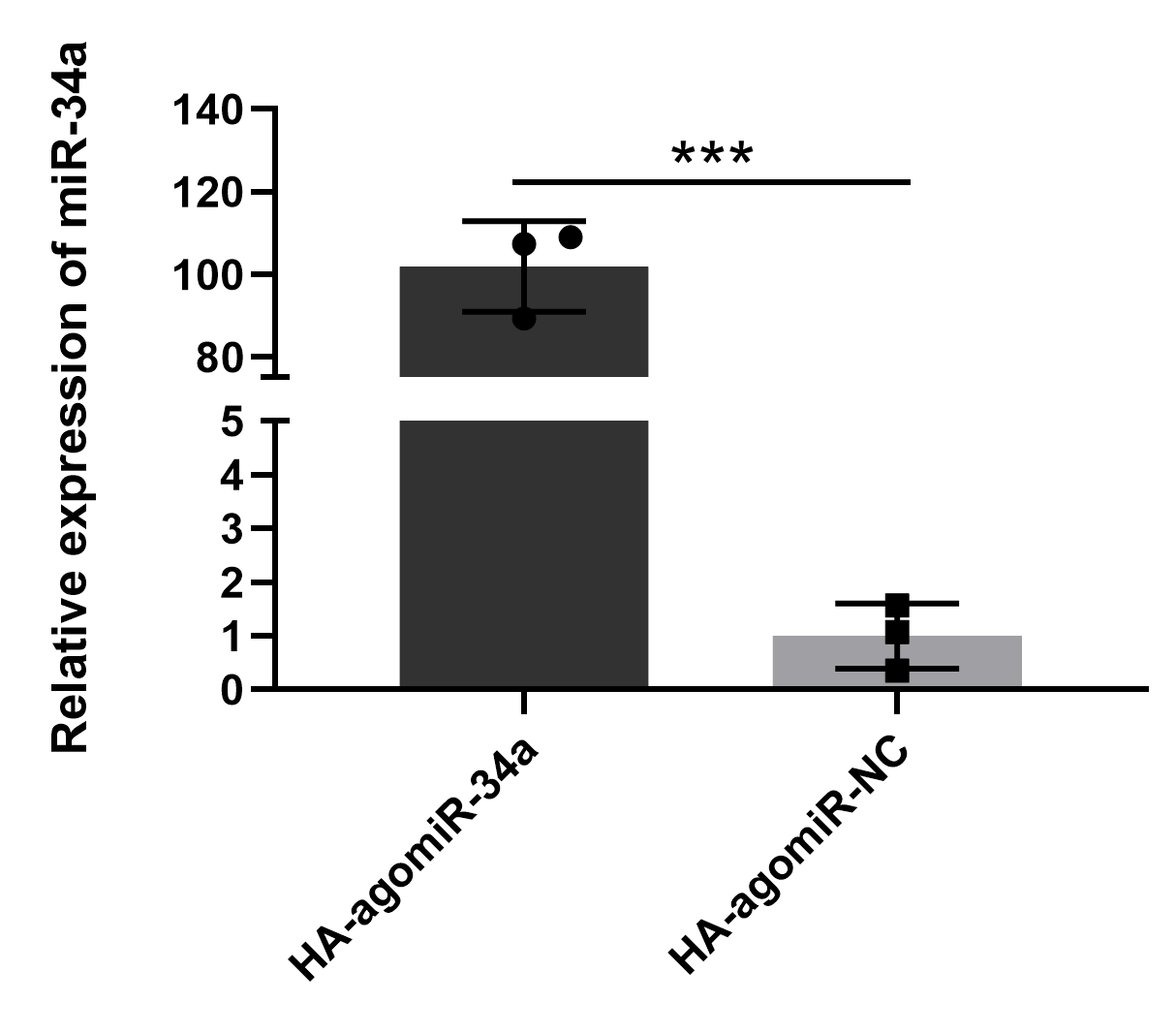


**Figure S1. Storable stability of miR-34a functionalized HA**. MiR-34a expression determined by qRT-PCR in BMSCs 48 hours after transfection. Note: HA-agomiR-34a was stored at 4°C for 90 days. Data are shown as mean ± SD, n=3; ***p<0.001

**Figure S2**


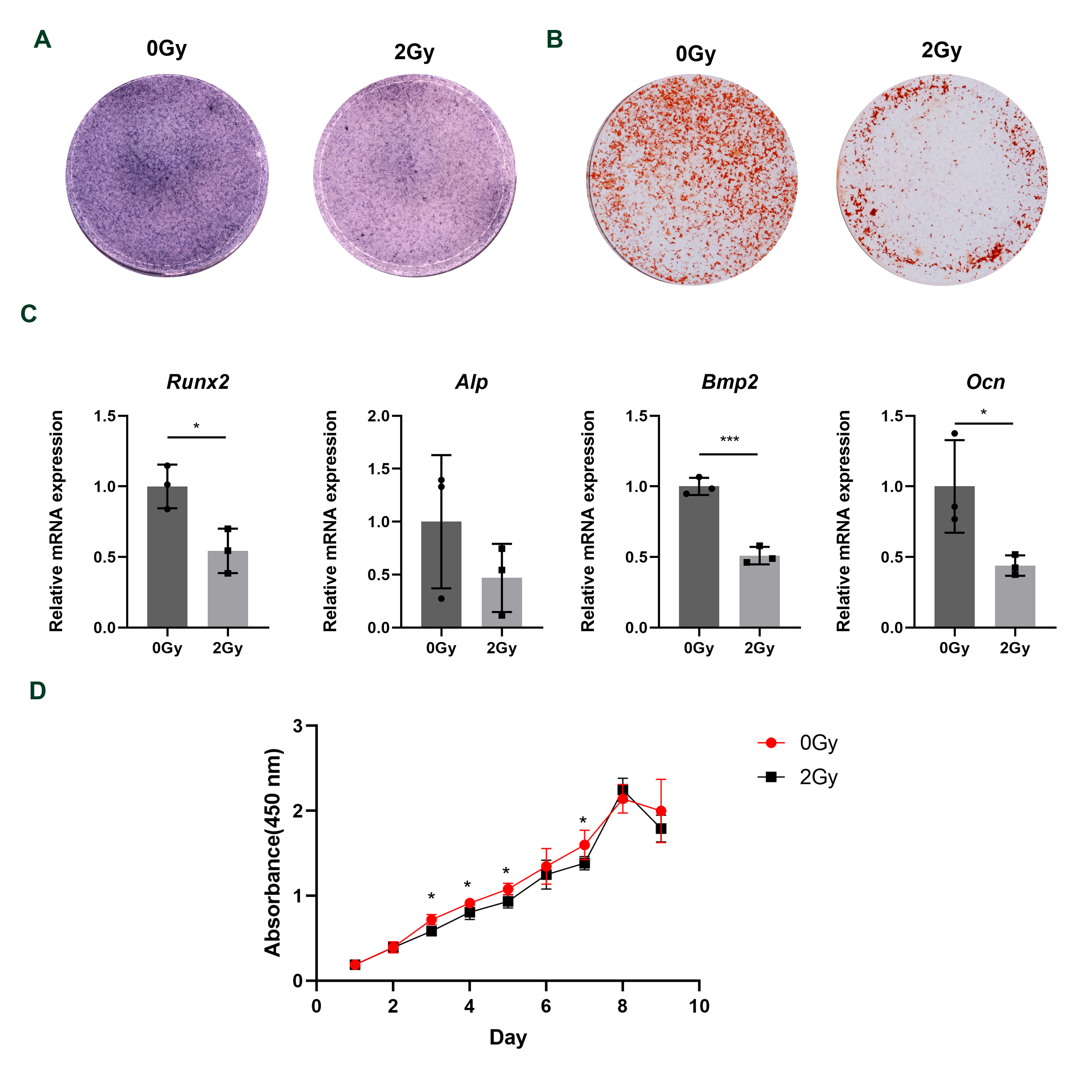


**Figure S2. The osteoblastic differentiation and proliferation of non-irradiated and 2 Gy irradiated BMSCs**. A: ALP staining after 7 days of osteogenic induction. B: Alizarin red staining after 21 days of osteogenic induction*.* C: Gene expression of *Runx2, Alp, Bmp2,* and *Ocn* after 14 days of osteogenic induction. Data are shown as mean ± SD, n=3; *p<0.05, ***p<0.001. D: The proliferation of BMSCs tested by CCK8. Data are shown as mean ± SD, n=5; *p<0.05, ***p<0.001.

**Figure S3**


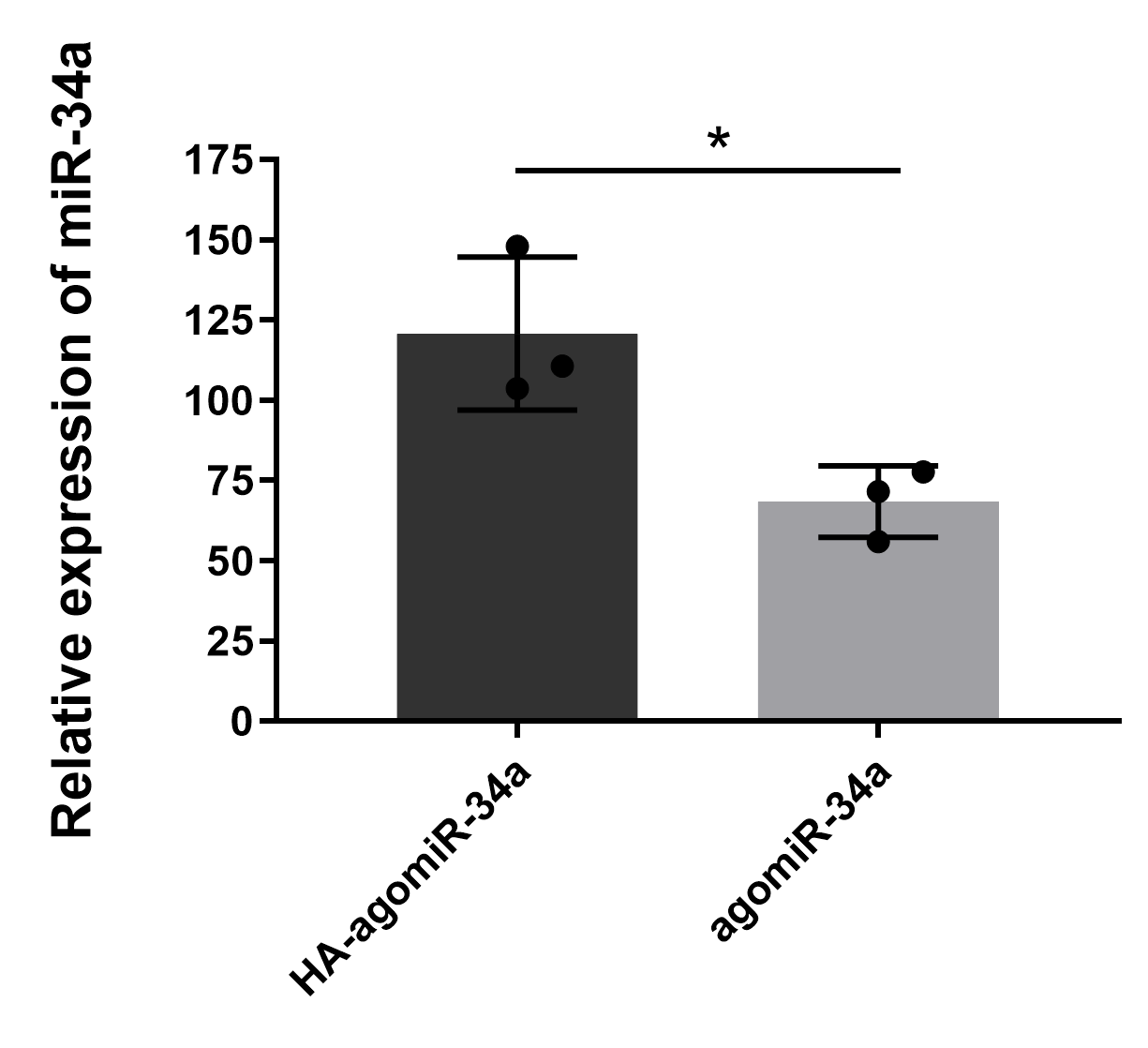


**Figure S3. Comparison of miR-34a expression between HA-agomiR-34A mediated transfection and conventional transfection.**

Notes: BMSCs were cocultured with HA-agomiR-34A or transfected conventionally with Lipofectamine2000/agomiR-34a complexes. The amount of Lipofectamine 2000 and agomiR-34a was identical in the two groups. Expression of miR-34a was assessed by qRT-PCR and normalized to the control group. The control group for HA-agomiR-34A was HA-agomiR-NC. The control group for agomiR-34a were agomiR-NC. Data are shown as mean ± SD, n=3; *p<0.05.

**Figure S4**


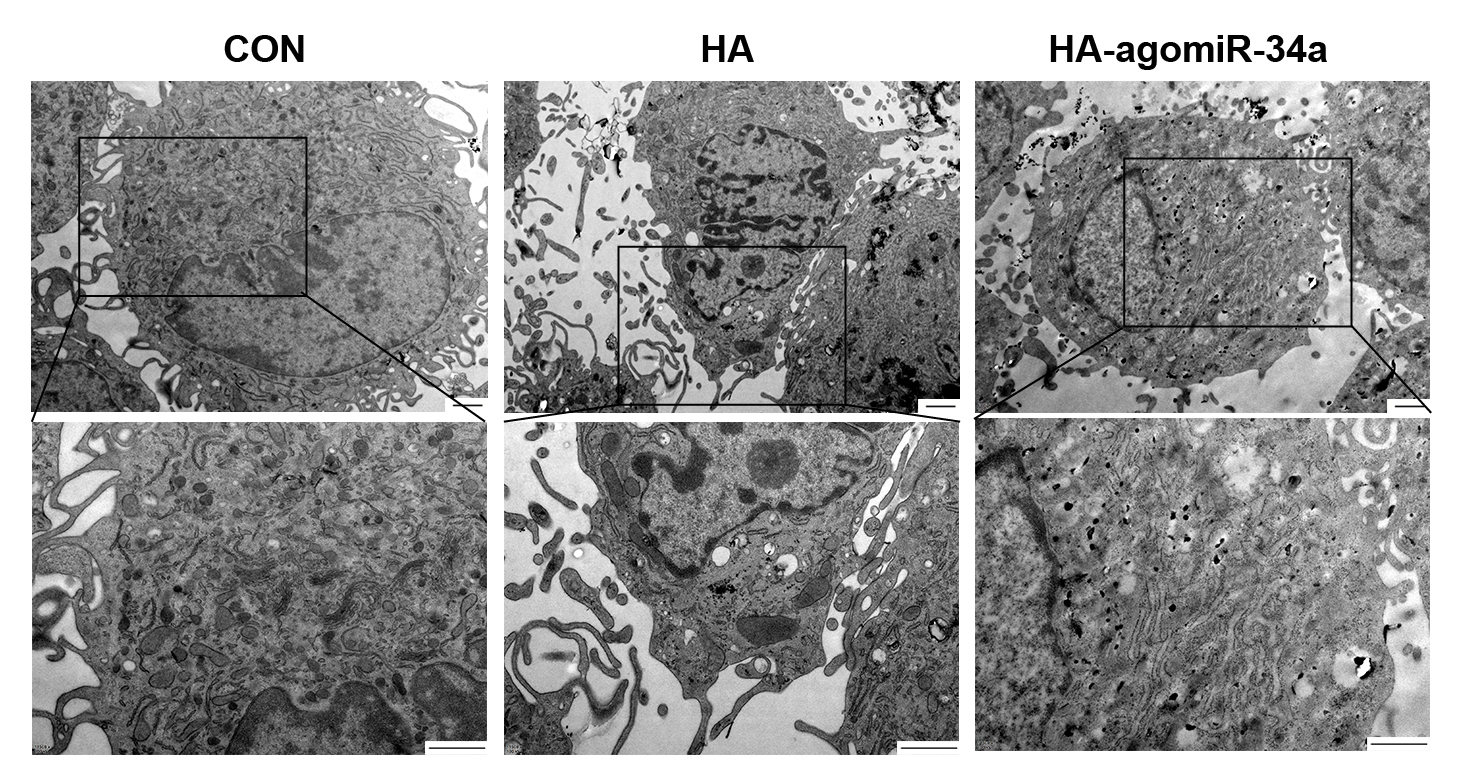


**Figure S4 The internalization of nanoparticles.** Representative TEM images of BMSCs and BMSCs cultured with HA or HA-agomiR-34a; bar=10μm.
